# Burden and determinants of malnutrition among pregnant women in Africa: A systematic review and meta-analysis

**DOI:** 10.1371/journal.pone.0221712

**Published:** 2019-09-06

**Authors:** Hanna Demelash Desyibelew, Abel Fekadu Dadi

**Affiliations:** 1 Department of Public Health Nutrition, School of Public Health, College of Medicine and Health Sciences, Bahir Dar University, Bahir Dar, Ethiopia; 2 Department of Epidemiology and Biostatistics, Institute of Public Health, College of Medicine and Health Sciences, University of Gondar, Gondar, Ethiopia; 3 College of Medicine and Public Health, School of Public Health, Flinders University, Bedford Park, Australia; University of Campus Biomedico, ITALY

## Abstract

**Background:**

Malnutrition in pregnancy remains unacceptably high across all regions of Africa though promising progresses have been made globally. Primary studies might not be sufficient to portrait a comprehensive picture of malnutrition during pregnancy and its main risk factors. Therefore, we intended to review the burden of malnutrition, for this specific review implies to protein energy malnutrition, during pregnancy in Africa to present its magnitude and determinant factors.

**Methods:**

We did a systematic review of observational studies published from January 1/2008 to January 31/2018. The CINAHL(EBSCO), MEDLINE (via Ovid), Emcare, PubMed databases and Google scholar were searched. Articles quality was assessed using the Newcastle-Ottawa Scale and studies with fair to good quality were included. We pooled malnutrition prevalence and an odds ratio estimates for risk factors after checking for heterogeneity and publication bias. This review has been registered in Prospero with a protocol number CRD42018114949.

**Result:**

23 studies involving 20,672 pregnant women were included. Using a random effect model, the overall pooled prevalence of malnutrition among pregnant women in Africa was 23.5% (95%CI: 17.72–29.32; I^2^ = 98.5%). Based on the current review pooled odds ratio finding; rural residency (POR = 2.6%; 95%CI: 1.48–4.65; I2 = 0%), low educational status of partners (POR = 1.7%; 95%CI: 1.19–2.53; I2 = 54.8%), multiple pregnancy (POR = 2.15%; 95%CI: 1.27–3.64; I2 = 0%) and poor nutritional indicators (POR = 2.03%; 95%CI: 1.72–2.4, I2 = 0%) were positively determine maternal malnutrition. On contrary, better household economic status (POR = 0.47%; 95%CI: 0.36–0.62; I2 = 24.2%) negatively determine maternal malnutrition.

**Conclusion:**

A significant number of the pregnant population in Africa are suffering of malnutrition, above 10% of the standard acceptable malnutrition rate. Thus, efforts should be renewed to ensure a proper and widespread implementation of programs that would address issues identified in the current review to reduce the burden of malnutrition.

## Background

Issues related to maternal malnutrition, for the case of this review implies protein energy malnutrition, have continuously posed a tremendous challenge in low-income countries in the face of extensive advancement in global economic growth [1–3]. Maternal malnutrition remains unacceptably high across regions in South-central and Southeast Asia and Sub-Saharan Africa [1, 4]. According to the 2018 World Health Organization (WHO) African region data, between 2000 and 2015, nine countries in Africa had a prevalence rates above 15%. Maternal underweight exceeds 20% in Ethiopia, Madagascar and Senegal while the lowest rates of underweight among women are found in Benin, Cameroon, Ghana, Lesotho, Rwanda, Swaziland, and Togo [5].

Globally, hunger and malnutrition reduce a Gross Domestic Product (GDP) of a given country by 1.4–2.1 trillion US Dollar a year. Similarly, malnutrition costs between 3 and 16% annual GDP of the 54 African countries and for mentioning few as example: Ethiopia 16.5%, Malawi 10.3%, Rwanda 11.5%, and Burkina Faso 7.7%. [2, 6, 7]. Maternal malnutrition also plays a central role in influencing maternal, neonatal, and child health outcomes [8]. New evidence indicates the importance of maternal nutrition for the first 2 years of child life for prevention of stunting and subsequent obesity and non-communicable diseases in adulthood [8, 9]. Similarly, poor maternal nutrition prior to and during pregnancy is strongly linked with increased risk of maternal anemia, mortality, and adverse birth outcomes such as Low Birth Weight (LBW) and Preterm Birth (PTB) [2, 10] though the explanation for this link has been very complex [2, 8].

Establishing a national nutrition targets to improve the national nutritional status is therefore a priority area that might affect the focus of nutrition policy of countries. As a target for 2030, different documents have been propelled to address maternal and child malnutrition. The World Health Assembly and Sustainable Development Goal documents are clearly highlighted the importance of introducing nutrition policy that gives due attention to maternal and child nutrition by its member countries [1, 6]. Implementation of this ambitious nutritional targets set in the national and global documents need to be supported with an endless and up-to-dated evidences. Thus, we conducted this systematic review and meta-analysis that aimed to summarize studies that have been conducted on malnutrition during pregnancy in Africa to generate a comprehensive data for professionals and decision makers so as to support progresses in policy implementation.

## Methods

### Data sources and search strategies

The primary outcome of this review was a protein energy malnutrition measured at any time of pregnancy. Observational studies published in Africa and written in English were searched systematically from January 1^st^, 2008 to December 31^st^, 2018 in the following databases and search Engines: CINAHL(EBSCO), MEDLINE (via Ovid), Emcare, PubMed, Google scholar and in addition, snow balling and retrieving references from a list of eligible studies were employed included.

#### Example of search strategy in PubMed

(((((nutritional status) OR malnutrition) OR underweight) OR under-nutrition)) AND (((pregnant women) OR pregnant mother) OR pregnancy) filters: Observational Study; Publication date from 2008/01/01 to 2018/12/31; Humans; English

#### Inclusion criteria

Studies that assessed malnutrition during pregnancy using the measurement of body mass index (BMI) and Mid Upper Arm Circumference (MUAC) were included.

#### Exclusion criteria

we excluded studies conducted in high or low risk pregnant population such as those living in refugee camps, Human Immune Virus (HIV) patients, or if the study has been conducted in any other restricted populations like adolescent pregnant population as prevalence studies conducted in such restricted population might not represent the general population. And more, studies published other than English language.

### Data extraction

First, a systematic searching was conducted through all identified data bases, search engines, and additional references were retrieved from other published articles. Second, studies conducted before 2008, conducted out of African countries, and unrelated articles based on their title were excluded. Third, those potentially eligible for inclusion were imported to Endnote version 7 and exact duplicates were removed. Fourth, two independent reviewers were done abstract and full-text review and data abstraction. In case of disagreement between the two reviewers, discussion was made to reach consensus. Lastly, the following data were extracted on structured data extraction form and presented using tables: Name of the leading author, year of publication, country in which the study was conducted, residence, study setting, study design, sample size, tool used to screen malnutrition, cut of point for screening tool, prevalence, odds ratio of adjusted associated factors with their 95% conficence intervals.

### Risk of Bias assessment

We used the Newcastle-Ottawa Scale (NOS) for assessing a quality of included studies. The NOS included 3 categorical criteria with a maximum score of 9 points. The quality of each study was rated using the following scoring algorithms: ≥7 points were considered as “good”, 2 to 6 points were considered as “fair”, and ≤ 1 point was considered as “poor” quality study. Accordingly, in order to improve the validity of this systematic review result, we only included primary studies with fair to good quality. We used the Meta-analysis Of Observational Studies in Epidemiology (MOOSE) statement for reporting our finding [11].

### Strategy for data synthesis

Prevalence of malnutrition and estimates for risk factors obtained from each study were pooled and determined as a single estimate. Before analysis, transforamtion of odds ratios were made. Publication bias was assessed using a visual inspection of funnel plots and Egger’s regression test. In the case of minor publication bias Tweedie and Duval’s Trim and Fill analysis was used as an adjustment. Heterogeneity between studies were explored using Higgins test where *I*^*2*^ statistic was calculated and reported. Pooled estimate from random effect model was reported in the case of significant heterogeneity and sub-analysis of estimates was done by country in which the study was conducted, income of the countries, study design, and measurement tools.

Risk factors obtained from each primary studies were thematically organized and their effect sizes were pooled accordingly. Sensitivity analyses was conducted for studies included in the meta-analysis [12, 13]. The Preferred Reporting Items for Systematic Reviews and Meta-Analyses (PRISMA) statement for reporting a systematic review and meta-analysis was used to clearly present the study inclusion, exclusion and reason for exclusion information in diagram. All statistical analyses were conducted in Stata 14 software. The protocol of this systematic review has been registered with the prospective register of systematic reviews (PROSPERO; registration number CRD42018114949).

## Result

### Study selection and characteristics

**Fig 1:**
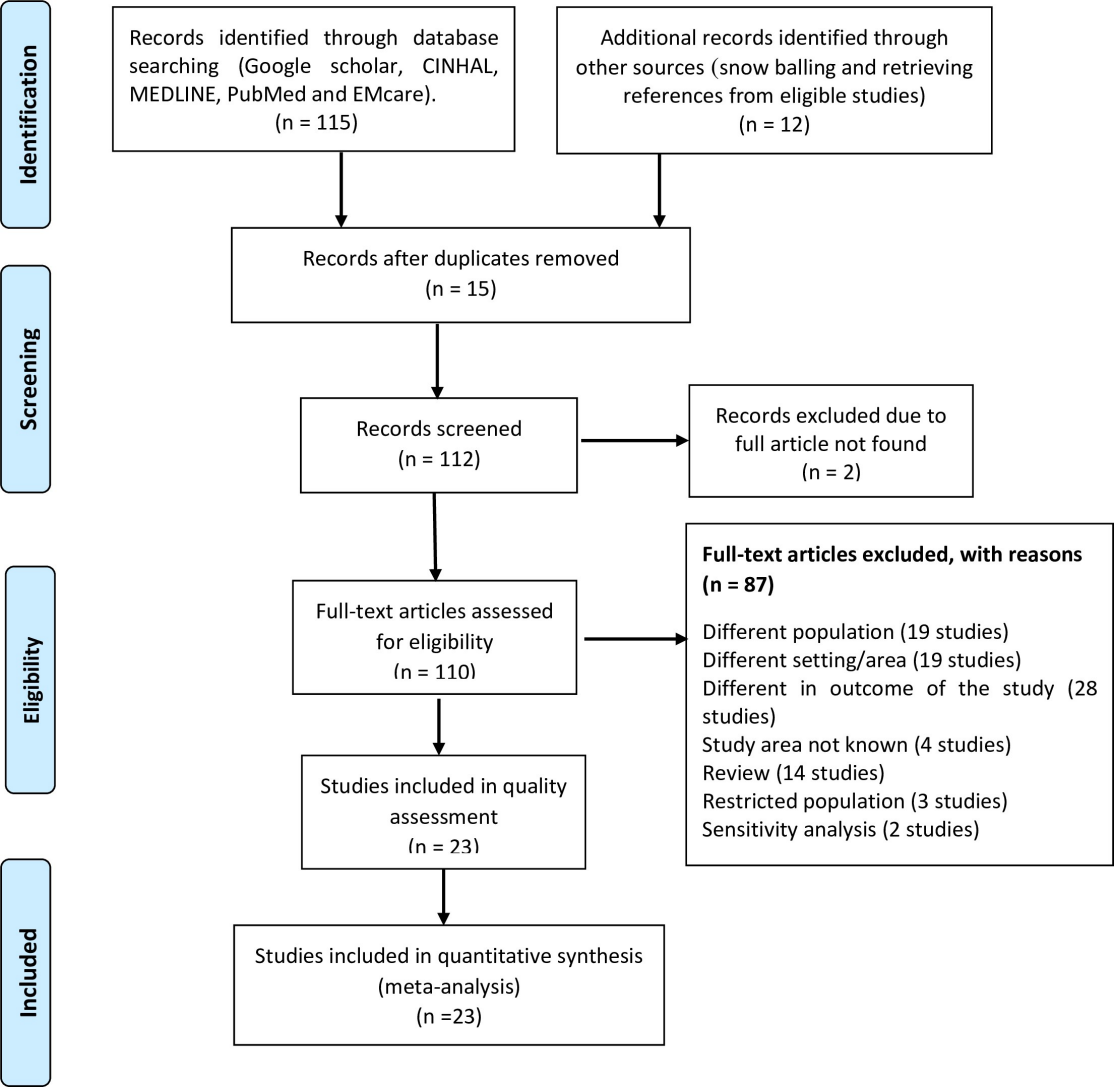
PRISMA statement presentation for systematic review and meta-analysis of malnutrition during pregnancy in African countries.

A total of 127 records were identified through database searching and 104 retrieved studies were omitted through step-by-step process for the following reasons: 15 studies were duplication [14–28], 14 were review studies [29–42], 1 was conducted in refugee camp [43], 2 recruited only adolescent pregnant mothers[44, 45], 4 had no full text [46–49], and the rest 66 articles had different study population [50–68], area [69–87], and outcome [14–22, 88–106]. (Fig 1). Lastly, the quality of 23 articles were assessed by using the NOS criteria. (Table 1)

**Table 1:** Characteristics of included study in systematic review and meta-analysis of studies conducted on malnutrition during pregnancy in African countries (N = 23).

S. No	Authors,year	Country, Income	Study design	Setting	Sample size	Residence	Measur ement	Cut off value	Prevalence	NOS result
**1.**	Siza JE.2008	Tanzania	Cross sectional	HI	3464	Rural-urban	BMI	<22	42.3	8
**2.**	Assefa N, *et al* 2012	Ethiopia	Cohort	HI	956	Semi-urban	MUAC	<23	47.2	4
**3.**	Regassa N. *et al*. 2012	Ethiopia	Cross sectional	community	1094	Rural	BMI	<18.5	28.1	8
		Ethiopia				Rural	MUAC	<22	31.4	
**4.**	Kuche D. et al. 2015	Ethiopia	Cross sectional	community	153	Rural	MUAC	<22	9.2	7
**5.**	Moges M et al. 2015	Ethiopia	Cross sectional	community	417	Rural-urban	MUAC	<21	35.5	6
**6.**	Kedir H, et al 2016	Ethiopia	Cross sectional	community	1731	Rural	MUAC	<22	19.1	8
			Cross sectional				BMI	<19.8	23.3	
**7.**	Kiboi W. et al 2016	Kenya	Cross sectional	HI	2454	Rural-urban	MUAC	< 23	19.3	6
**8.**	Abebe FM, et al. 2018	Ethiopia	Cross sectional	community	316	Rural-urban	MUAC	<21	31.8	7
**9.**	Kumera G, et al 2018	Ethiopia	Cross sectional	HI	409	Rural-urban	MUAC	<22	16.2	8
**10.**	Nigatu M, et al. 2018	Ethiopia	Cross sectional	community	338	Rural-urban	MUAC	< 21	28.6	8
**11.**	Shenka A, et al 2018	Ethiopia	Cross sectional	HI	387	Rural-urban	MUAC	<22	18.2	8
**12.**	Padonou SG, et al 2018	Benin	Cross sectional	HI	526	Rural-urban	BMI	<20	29.8	6
**13.**	Landis S, et al 2009	DRC	Cohort	HI	182	Rural-urban	MUAC	<23	14.0	4
						Rural-urban	BMI	<19.8	11.0	
**14.**	Rayis DA, et al 2010	Sudan	Cross sectional	HI	1690	Rural-urban	BMI	< 19.9	5.5	8
**15.**	Chigbu C, et al 2011	Nigeria	Cohort	HI	3167	Rural-urban	BMI	<18.5	3.0	4
**16.**	Oni OA, et al 2012	Nigeria	Cross sectional	HI	405	Rural	MUAC	< 22.9	21.7	7
**17.**	Ouédraogo S, et al 2012	Benin	Cross sectional	HI	1005	Semi-urban	BMI	<22	44.3	7
**18.**	Melku M, et al 2014	Ethiopia	Cross sectional	HI	302	Rural-urban	BMI	< 20	9.9	7
**19.**	Elmugabil A, et al 2017	Sudan	Cross sectional	HI	388	Rural-urban	BMI	<18.5	4.4	7
**20.**	Kumera G, et al 2018	Ethiopia	Cross sectional	HI	234	Rural-urban	MUAC	<22	41.0	8
**21.**	Kefiyalew F, et al 2014	Ethiopia	Cross sectional	HI	258	Rural-urban	BMI	<18.5	11.6	7
**22.**	Derso T, et al2017	Ethiopia	Cross sectional	HI	348	Rural-urban	MUAC	<23	35.8	8
**23.**	Tadesse SE, et al 2017	Ethiopia	Cross sectional	HI	448	Urban	MUAC	<23	30.4	8

Twenty three studies (n = 20,672) with 26 estimates had fair to good quality and included in the final review [23–28, 107–123]. The smallest study was a study by *Kuche D*. *et al*. *2015* (included only 153 samples) while the biggest study was a study by *Siza JE*, *et al*.*2008* that included 3,464 samples. A mean sample size for included studies was 2,131. The smallest prevalence of malnutrition was estimated by Chigbu C, et al (3.0%) and the largest prevalence was estimated by Assefa N, et al (47.2%). The majority of included studies were obtained from Ethiopia (14 studies) [24, 25, 27, 107, 108, 110–112, 114, 115, 117, 118, 122, 123], where the rest were from Eastern Africa (Kenya, Tanzania), Western Africa (Nigeria, Benin), Northern Africa (Sudan), and Central Africa (Democratic Republic of Congo). Twenty studies were cross sectional, six studies were community based, and 17 studies were conducted in a rural-urban setting. Regarding measurement of nutritional status, three studies used both MUAC and BMI, eight studies used BMI, and twelve studies used MUAC. According to the NOS criteria, six studies fail in fair quality category [108, 109, 113, 116, 117, 120] while the rest seventeen fail [23–28, 107, 110–112, 114, 115, 118, 119, 121–123] in good quality category. (Table 1)

### Synthesized outcomes

**Fig 2:**
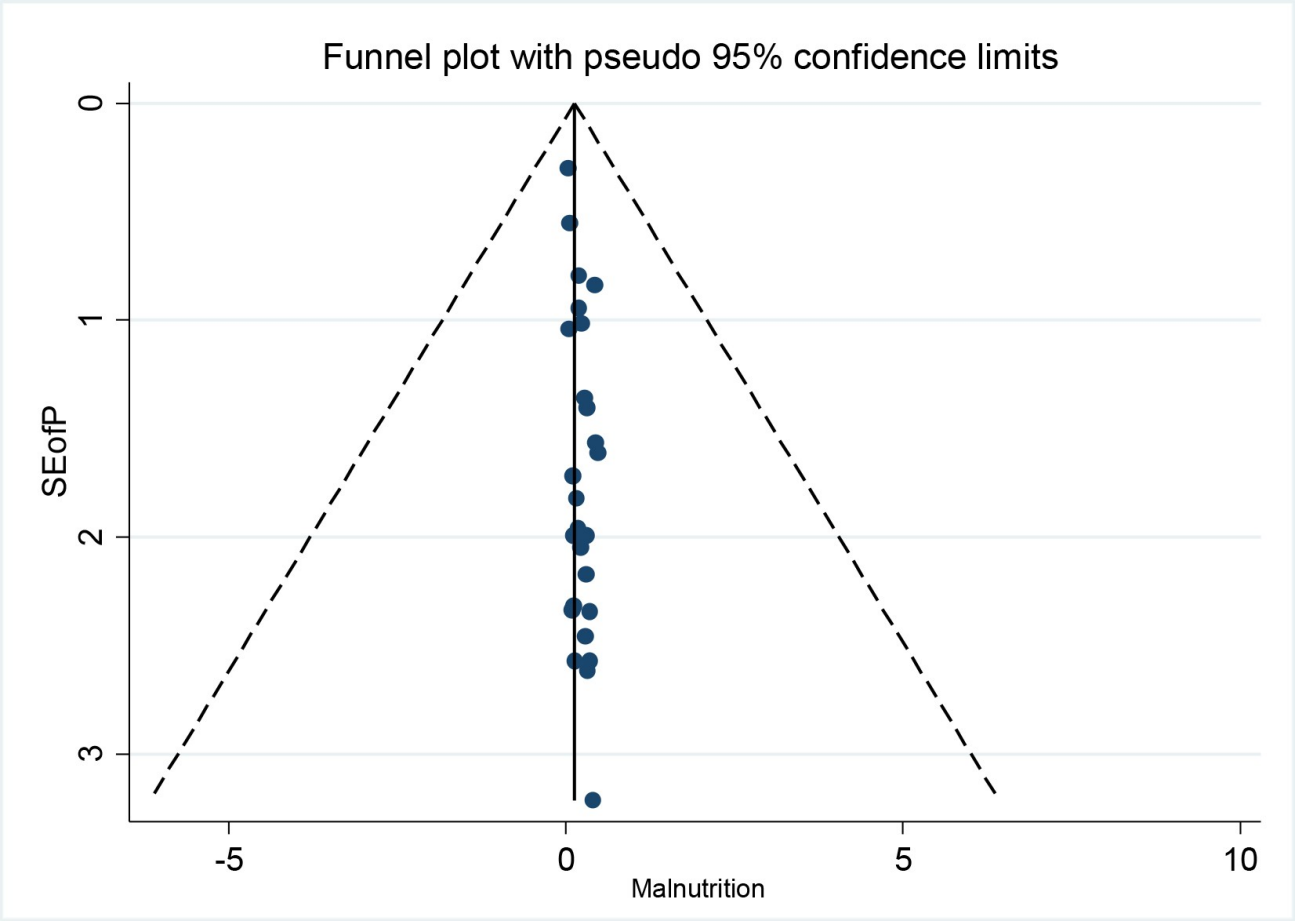
Funnel plot presented the visual inspection of publication bias for systematic review and meta-analysis of malnutrition during pregnancy in African countries.

Evidence of high heterogeneity between the included studies is observed as indicated by the Q and I^2^ statistics. The asymmetrical visual inspection of the Funnel plot and the quantitative Egger’s regression test significant value (P<0.001) evidenced a presence of publication bias in the current systematic review.(Fig 2)

**Fig 3:**
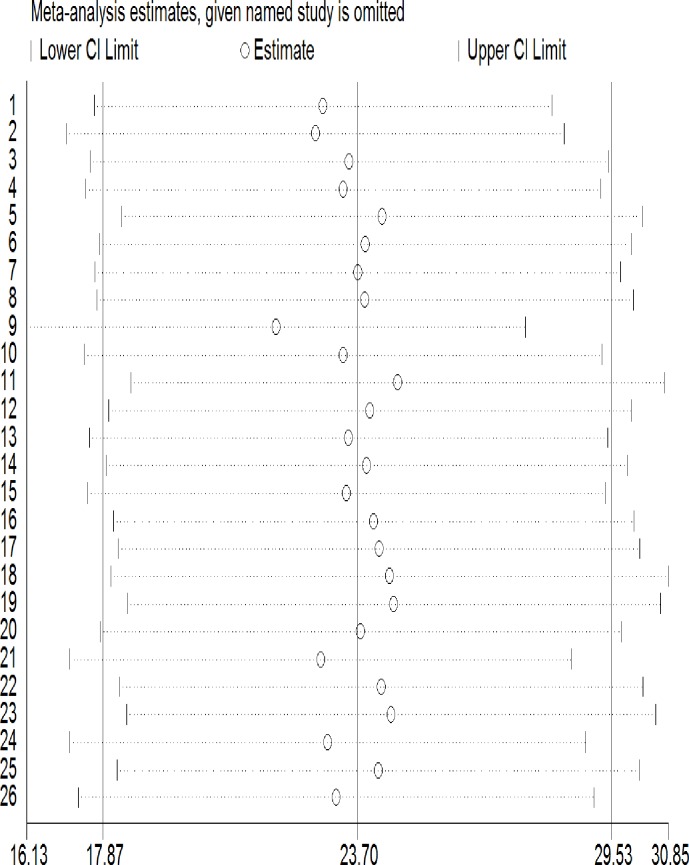
Sensitivity analysis for studies included in a systematic review and meta-analysis of malnutrition during pregnancy in African countries.

In Africa, maternal malnutrition was estimated to be 23.5% (95%CI: 17.7–29.3, I^2^ = 98.5%) based on pooling of 23 primary studies conducted in different parts of Africa. Because of a significant heterogeneity we did a Meta regression to identify the possible source. We found that a type of measurement used and a country in which the study was conducted explained the observed heterogeneity by seven and nine percent, respectively. Thus, we conducted and reported a pooled prevalence from a sub-analysis obtained from a random effect model. In our sub-analysis, the pooled prevalence of malnutrition was higher in Ethiopia (PP = 26%; 95%CI: 20.9–29.3) compared to African pooled data (PP = 23.5, 95%CI: 17.7–29.3, I^2^ = 98.5%) and other African countries (PP = 19.5; 95%CI: 10.0–29.0). Similarly, malnutrition prevalence was higher among studies used MUAC as a screening tool (PP = 26.5; 95%CI; 21.5, 31.6) compared to those used BMI (PP = 19.4; 955CI; 10.2, 28.6). (Table 2). We did a sensitivity analysis for studies included and two studies found highly influential and were excluded from the final analysis [12, 13]. (Fig 3)

**Table 2:** Malnutrition during pregnancy, sub-group meta-analyses (N = 23, random effect model).

Variables	Number of studies	Sample size	Pooled estimate, 95%CI
Country in which the study was conducted			
Ethiopia	14	7,391	26.00(20.9,29.3)
Other African countries	9	13,281	19.52(10.0,29.0)
Measurement			
BMI	11	11,894	19.4(10.2,28.6)
MUAC	15	9,872	26.5(21.5,31.6)
<21	3	1071	32.0(28.0,36.0)
<22	6	4008	20.3(13.8,26.9)
<23	6	4,793	28.1(17.6,38.5)
Overall pooled estimate	23	20,672	23.5(17.7,29.3)

**Fig 4:**
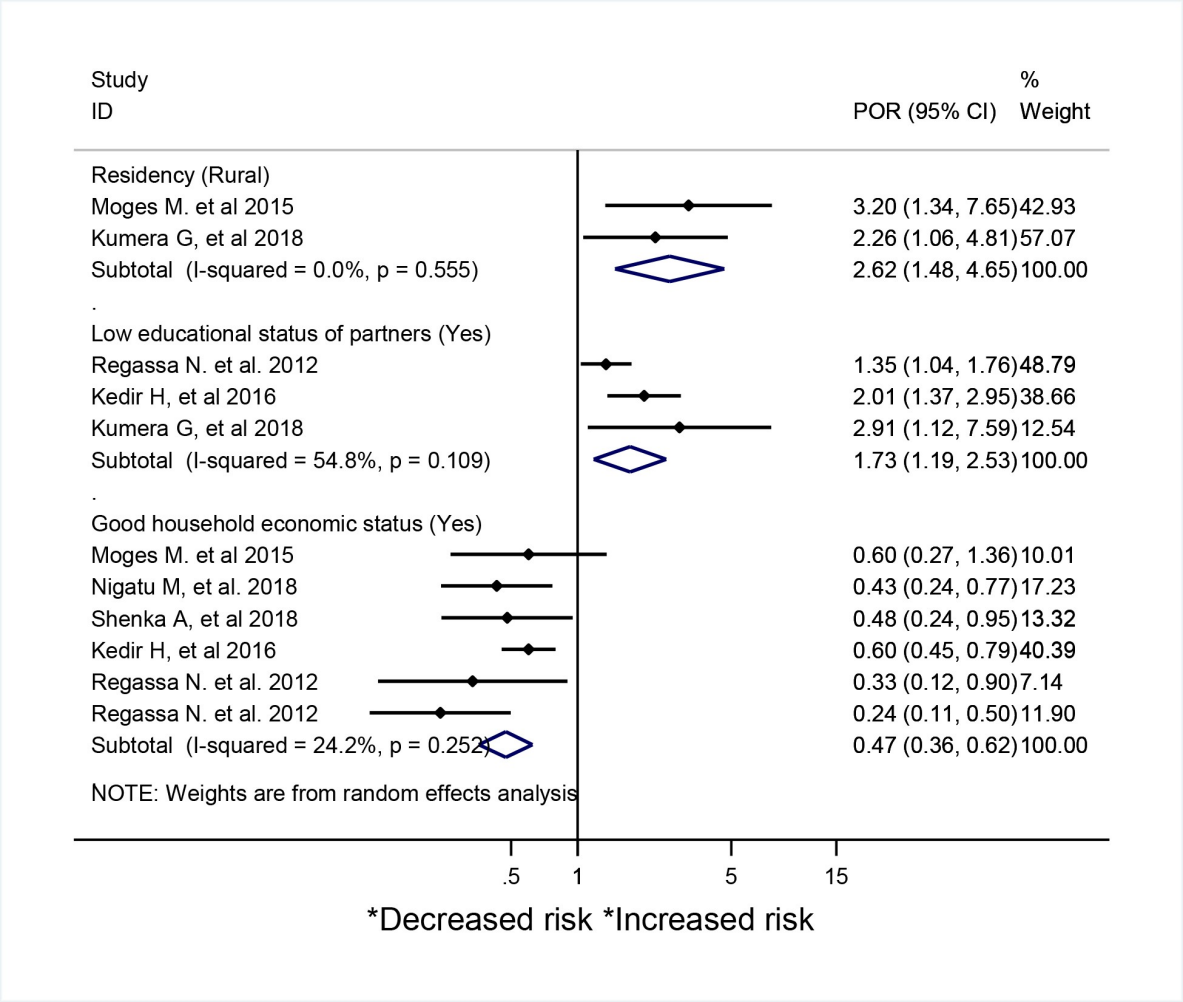
A Systematic review and meta-analysis of risk factors associated with malnutrition among pregnant mothers in Africa.

To identify the contributing risk factors associated with maternal malnutrition, we thematically organized risk factors reported in each primary studies and finally pooled their odds ratio estimates. Thus, the first factor which was reported repeatedly in two primary good quality studies were residency, the odds of being malnourished was 2.6 times (95%CI: 1.48–4.65; I^2^ = 0%) higher among rural resident compared to urban [24, 117]. The pooled effect of Meta-analysis, as well as the consistent findings from each good quality primary studies, confirmed that low educational status of partners increases the risk of maternal malnutrition by 1.7% (95% CI: 1.19–2.53; I^2^ = 54.8%) [24, 27, 111]. (Fig 4)

The other important factor which associated with decreased odds of malnutrition was family’s better economic status and the odds of being malnourished was 0.47 (95%CI: 0.36–0.62; I^2^ = 24.2%) times lower among pregnant mothers who are living in household with better economic status (that explained by better land access or land owned by households, household food security, better food access, high income and possessing livestock’s) in five studies [27, 111, 117, 118]. (Fig 4)

**Fig 5:**
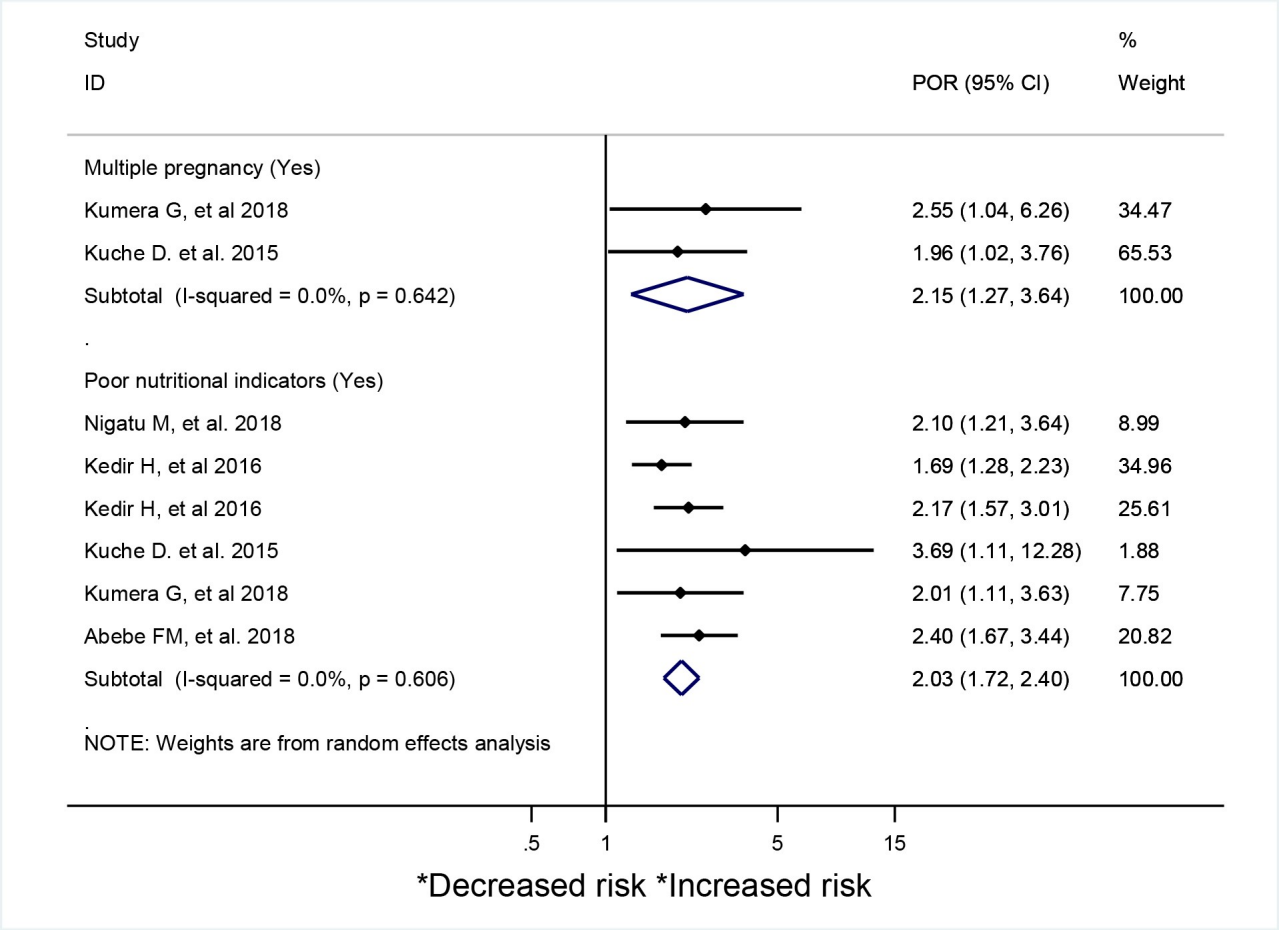
A systematic review and meta-analysis of factors associated with malnutrition during pregnancy in Africa.

Pregnant mothers with a history of multiple pregnancy (POR = 2.15; 95% CI: 1.27–3.64; I^2^ = 0%) were more likely to be malnourished based on an estimates obtained from two good quality primary studies [24, 114] (Fig 5). Poor nutritional indicators (that explained by having poor feeding practice, lack of knowledge on proper feeding during pregnancy, being anemic, having poor dietary diversity score (DDS), and not eating cereal foods) were positively and repeatedly associated with increased odds of maternal malnutrition (POR = 2.03; 95% CI: 1.72–2.4; I^2^ = 0%) in five studies with good quality [24, 27, 107, 111, 114, 117, 118, 122]. (Fig 5)

## Discussion

The global estimate of maternal malnutrition during pregnancy appears to be decreasing in almost all regions of the globe except in Africa where the number of pregnant mothers with malnutrition has been increasing steadily over time. This information could be witnessed by a meta-analysis done across 27 Sub-Saharan African countries [124] and our current updated review. The rate of maternal malnutrition was reported as 16.6% in previous review while we found 23.5% in our current review and both estimates exceed 10%, which is a cut-off score for declaring maternal malnutrition as a major public health problem [3, 124, 125].

Maternal malnutrition is associated with an increased risk of maternal morbidity and mortality with a number of adverse pregnancy outcomes like low birth weight and preterm birth, which could also be associated with a high risk of new born morbidity and mortality [10, 124, 126]. In agreement with this evidence, it would not be wrong to predict that 23.5% of pregnant mothers in Africa are living with malnutrition problem and they might have been suffering from pregnancy complications and adverse birth outcomes associated with their nutritional problem. However, the World Health Assembly is targeted to reduce LBW by 30% and stunting and women anemia by 50% by 2030 both globally and regionally in Africa [1]. Nevertheless, continuingly, Africa is facing a rising number of malnourished women with a small reduction in number of LBW infants [1, 3]. Thus, since data and the literature review supports a strong association between maternal malnutrition and adverse pregnancy and birth outcomes like PTB and LBW [1, 10], intervention plans focusing on enhancing nutritional status of women in reproductive age and during pregnancy would help in achieving the above-mentioned targets of 2030.

We also identified the following risk factors for this high malnutrition prevalence that might help to take focused interventions: Rural residency, low educational status of partners, low economic status of households, having multiple pregnancy and poor nutritional indicators.

In line with a review conducted in Sub-Saharan countries [127], we also found that the odds of malnutrition was found to be higher in pregnant mothers living in rural areas. Pregnant mothers living in rural areas had a 2.6 times higher chance of being malnourished compared to their urban counterparts. It is true that place of residence usually determines people's life-styles, income, social and cultural activities, and most notably their health conditions and nutrition.

Low educational status of the partners was also significantly influenced maternal nutrition. We found that the odds of malnutrition was 1.7 times higher among pregnant mothers having a low educational attainment, which is consistent with other reports [128, 129]. This is might be true that educated partners are cautious about their family nutrition than uneducated partners. On the other side, better educational attainment of partners might also be correlated with earning a better income to ensure their household food security [2].

Our pooled result suggested that pregnant women who lived in households with better economic status had a 53% lower odds of being malnourished. This finding is consistent with a report obtained from the Global Burden of Maternal and Child malnutrition [129]. It is true that women from household with better economy are more likely to access food and nutrient rich sources for their daily requirement to decreases their risk of malnutrition [2, 128].

We found that women with two or more pregnancies had a 2.15 times increased chance of being malnourished than a women with a single pregnancy. It is true that physiological stress during pregnancy demands extra nutrient that could put pregnancy and lactation time at higher risk for malnutrition than other women’s life time. On top of this, when the women enter in to vicious cycle of too many closely spaced pregnancies, her tissues becomes depleted and she will be highly vulnerable for malnutrition [129, 130].

Our review found a significant correlation between poor nutritional indictors, as explained above, and maternal malnutrition in good quality primary studies. Women who had poor nutritional indicators were 2 times higher to be malnourished. Poor diet with lack of nutrient supplementation during pregnancy would worsen maternal malnutrition as indicated in the current and previous reviews [128, 130]. Similar to our review finding and an existing literature, high DDS is linked with decreased maternal malnutrition rate and has been also used as a proxy indicator for quality diet and adequate energy intake [128].

We found a conflicting ideas regarding the relationship between poor DDS and maternal malnutrition in the literature: A study conducted by *Nigatu M*, *et al*. showed that high DDS was associated with decreased odds of maternal malnutrition [118] and on contrary *Shenka A*, *et al*. reported the odds of maternal malnutrition that increased with high DDS [122]. However, in addition to our review finding and an existing literature, high DDS is linked with decreased maternal malnutrition rate and has been also used as a proxy indicator for quality diet and adequate energy intake [128].

## Limitation

As far as our reading covers, this is the first comprehensive review conducted in Africa searching through a broad data bases on malnutrition in pregnancy and reporting its comprehensive picture. However, a high level of heterogeneity as a result of using different anthropometric measurement for screening malnutrition, high discrepancy in prevalence of malnutrition, and under representation of all countries might affect the overall generalizability of the review to all African countries. However, the issue of heterogeneity across studies and lack of a sufficient number of studies from African countries has been addressed by using a random effect model for pooling the estimates. The random effects model takes into considerations any heterogeneity inherent in the meta-analysis and tends to give a more conservative estimate. The other limitation of this review was not including studies published in non-English language.

## Conclusion

Our systematic review and meta-analysis has estimated a high prevalence of malnutrition during pregnancy in Africa, which is also consistent irrespective of other observed source of variations. These unacceptably high maternal malnutrition estimates in Africa stressed the need for priority interventions targeted to improve maternal nutrition during pregnancy. Investing on maternal malnutrition is also a key strategy to reduce LBW, PTB, and to break its inter-generational effect. Apart from socio-demographic and economic factors, maternal malnutrition has been affected by inadequate nutrient intake or poor nutritional indicators and multiple pregnancies.

An optimal nutrient intake by the mothers is essential to meet both maternal and fetal requirements and reduce adverse health consequences in addition to spaced pregnancy. Although several nutritional intervention programs have been introduced to improve maternal nutrition globally and regionally, the problem has been increasing. Efforts should be renewed to ensure a proper and widespread implementation programs in order to significantly reduce its burden across African countries.

## Supporting information

S1 Checklist PRISMA statement presentation for systematic review and meta-analysis of malnutrition during pregnancy in African countries. (DOCX)

## Abbreviations

BMI: Body Mass Index
DDS: Dietary Diversity Score
DRC: Democratic Republic of Cong
EDHS: Ethiopian Demographic and Health Survey
GDP: Gross Domestic Product
HI: Health Institution
LBW: Low Birth Weight
MUAC: Mid-Upper Arm Circumference
NCDs: None Communicable Diseases
NNP II: National Nutrition Program II
NOS: Newcastle Ottawa Scale
PTB: Pre-Term Birth
